# Identification of distinctive physiological and molecular responses to salt stress among tolerant and sensitive cultivars of broccoli (*Brassica oleracea* var. *Italica*)

**DOI:** 10.1186/s12870-021-03263-4

**Published:** 2021-10-25

**Authors:** Sergio Chevilly, Laura Dolz-Edo, Luna Morcillo, Alberto Vilagrosa, José Manuel López-Nicolás, Lynne Yenush, José M. Mulet

**Affiliations:** 1 Instituto de Biología Molecular y Celular de Plantas, Universitat Politècnica de València-Consejo Superior de Investigaciones Científicas, 46022 Valencia, Spain; 2 Fundación Centro de Estudios Ambientales del Mediterráneo, Joint Research Unit University of Alicante – CEAM, University of Alicante, 03080 Alicante, Spain; 3 Departamento de Bioquímica y Biología Molecular-A, Facultad de Biología, Universidad de Murcia, 30100 Murcia, Spain

**Keywords:** Salt stress, Broccoli, Molecular markers, Metabolomics, Crop improvement, Krebs Cycle, Amino acids, Anaplerotic reactions

## Abstract

**Background:**

Salt stress is one of the main constraints determining crop productivity, and therefore one of the main limitations for food production. The aim of this study was to characterize the salt stress response at the physiological and molecular level of different Broccoli (*Brassica oleracea* L. var. *Italica* Plenck) cultivars that were previously characterized in field and greenhouse trials as salt sensitive or salt tolerant. This study aimed to identify functional and molecular traits capable of predicting the ability of uncharacterized lines to cope with salt stress. For this purpose, this study measured different physiological parameters, hormones and metabolites under control and salt stress conditions.

**Results:**

This study found significant differences among cultivars for stomatal conductance, transpiration, methionine, proline, threonine, abscisic acid, jasmonic acid and indolacetic acid. Salt tolerant cultivars were shown to accumulate less sodium and potassium in leaves and have a lower sodium to potassium ratio under salt stress. Analysis of primary metabolites indicated that salt tolerant cultivars have higher concentrations of several intermediates of the Krebs cycle and the substrates of some anaplerotic reactions.

**Conclusions:**

This study has found that the energetic status of the plant, the sodium extrusion and the proline content are the limiting factors for broccoli tolerance to salt stress. Our results establish physiological and molecular traits useful as distinctive markers to predict salt tolerance in Broccoli or to design novel biotechnological or breeding strategies for improving broccoli tolerance to salt stress.

**Supplementary Information:**

The online version contains supplementary material available at 10.1186/s12870-021-03263-4.

## Background

Nearly the 70% of earth’s surface is covered by salty water and about 10% of terrestrial habitats are affected by salt. In addition, anthropogenic global warming is altering the weather patterns and thus threatening agricultural production [1, 2]. Climate change is predicted to have two impacts that will worsen the salinization of land: the direct inundation of coastal areas by seawaters and the increased aridity [3]. With rising temperatures, the pressure on aquifers will increase and therefore also the chances of sea water infiltration due to the decrease in the phreatic level. In addition, repeated cycles of irrigation and evaporation combined with high levels of fertilization induce the accumulation of salts and thus soil salinization. Coping with salt is one of the major problems of agriculture and the presumed scenario will be much worse in the near future [4].

Halophytes (i.e. plants able to complete their life cycle under saline conditions that would prevent growth and/or reproduction in most species) are rare [5]. Less than 2% of flowering plants are halophytes [6], but this trait has emerged in at least 100 different angiosperm families [7]. From the evolutionary point of view, it seems that salt tolerance may be a macroevolutionary self-destructive trait, gained often but frequently lost by reversal or extinction [8]. From the horticultural point of view, breeding for salt tolerant crops has proven to be very difficult. The use of biotechnological crops and new breeding techniques is also very limited. In the literature, there is information on only two successful field trials of crops transformed with a gene able to increase salt tolerance [9]: barley expressing the *Arabidopsis thaliana* vacuolar H^+^-pyrophosphatase (*AVP1*) [10] and wheat expressing the vacuolar Na^+^/H^+^ antiporter gene *AtNHX1* from *Arabidopsis thaliana* [11].

The presence of salt in the irrigation water, mainly sodium chloride, causes two problems during plant development. First, in the soil, outside the plant, the salt is able to retain the water, preventing its absorption by the roots, thus making an effect similar to drought. On the other hand, when sodium cations enter the plant, they induce toxicity, as sodium displaces potassium cations, which are the main ions inside plant cells. This displacement interferes with different biochemical and physiological processes and can lead to cell death. Plants have developed complicated mechanisms to maintain ion homeostasis, mainly aimed at keeping the potassium concentration within the cell high, and the sodium concentration low [12].

The genus *Brassica* includes some important species of agronomic interest, including broccoli (*Brassica oleracea* L. var. *italica*, Plenck), a crop cultivated in temperate climates. Broccoli production has gained importance in recent years for its considerable nutritional value provided by its richness in bioactive compounds such as vitamins C and E, quercetin or kaempferol glycosides [13], as well as by the presence of glucosinolates, a group of about 120 molecules derived from amino acids [14] with a ß-D-thioglucose moiety [15]. In plants, glucosinolates have been shown to play a role in the defense against biotic stress [16], but in humans it has been proven that, apart from contributing to the characteristic flavor of broccoli, some compounds produced upon hydrolysis of glucosinolates, such as sulforaphane, may reduce the risk of lung, breast, gastric, prostate or kidney cancer [17, 18].

Broccoli is considered a crop moderately tolerant to salt stress. Its tolerance is higher than other common vegetables such as lettuce, onion, maize or carrot [19]. Salt stress in broccoli causes a two-phase growth decrease [20]. The first phase of the growth decrease is the consequence of salt surrounding the roots and the second phase results from the internal injury due to salt accumulation in leaves [21]. In general, the *Brassicaceae* family can tolerate salt stress by osmolyte accumulation, Na^+^ exclusion and a relatively high K^+^ retention ability [22]. Prior studies have also determined the changes in several physiological parameters upon salt stress, for instance a study comparing three commercial cultivars determined that leaf water potential only changes in the long term, and that the changes in the transpiration rate and stomatal conductance depend on the cultivar [23]. To our knowledge there are no studies in the literature comparing the hormone levels or the amino acid and metabolite contents of different cultivars with different levels of stress tolerance under control and salt stress conditions.

In the specific case of broccoli, the characterization of a cultivar as tolerant to salinity has always been made *a posteriori*, on the basis of the empirical evidence obtained in the field or the experience of farmers who have used certain cultivars. Many times, cultivars are used without having any information *a priori* on whether they are adapted to salty soils, with the consequent drop in production and loss of income for the farmer. It is also interesting to generate new cultivars able to withstand salt stress, as it has been demonstrated that salt stress enhances the nutritional quality of broccoli [24]. In addition, broccoli has considerable potential because of its susceptibility to biotechnological modifications, affording the possibility to design strategies to improve its resistance to various stresses [25]. Although it has been an active field of investigation, there is no commercial cultivar of broccoli generated using biotechnological tools in the market [26].

Previous studies carried out in our laboratory on non-model species, such as *Pinus halepensis* [27, 28], *Phaseolus vulgaris* [29] or *Vicia faba* [30], have demonstrated the usefulness of physiological or molecular markers to characterize abiotic stress tolerant cultivars. In the present paper, there is a characterization, using different physiological and chemical strategies, of two salt tolerant and two salt sensitive cultivars with the goal of identifying distinctive traits at the physiological or molecular level. Thus, the aim of our study was to determine if this study could observe differences in any physiological or molecular parameter among different cultivars and/or treatments. The identification of these traits will allow us to predict whether uncharacterized field cultivars are likely to be salt tolerant or salt sensitive and this information will also help to identify the limiting factors for broccoli salt tolerance. This knowledge will facilitate the breeding of new salt tolerance cultivars and will supply farmers with new cultivars with enhanced production in the context of climate change and help to provide consumers with broccoli with enhanced nutritional content [24].

## Results

### Physiological measurements

**Fig. 1:**
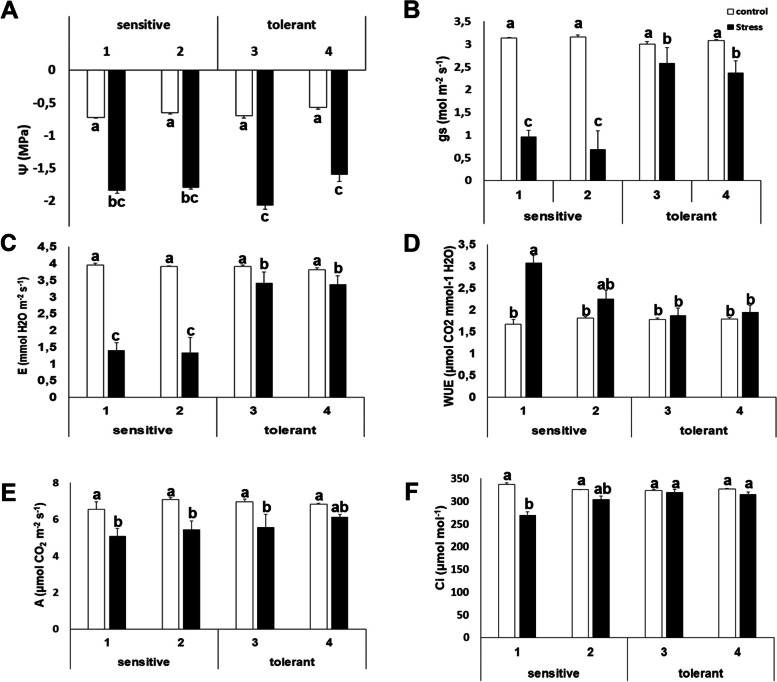
Physiological measurements. Water potential (Ψw) (**A**); stomatal conductance (gs) (**B**); transpiration (**E**) (**C**); instantaneous water use efficiency (WUE) (**D**); net photosynthesis (**A**) (**E**) and CO_2_ substomatical concentration (Ci) (**F**) of salt-sensitive and salt-tolerant cultivars under control (white bars) and stress (black bars) conditions. Data with different letters differ significantly (*p* < 0.05), as determined by Duncan’s MRT test (*n* = 5). Scale bars are the mean + Statistical Error (SE)

As expected, this study found a negative effect of salinity on water potential (Ψw) in both salt-tolerant and salt-sensitive cultivars. Values under salt stress significantly decreased by 2.5- to 3-fold compared to control values, indicating that plants were indeed stressed (Fig. 1A). The salinity treatment also had a negative effect on stomatal conductance (gs) and transpiration (E) (Fig. 1B and C). For both variables, values were lower for salt-sensitive cultivars, while results were about two-fold higher in salt-tolerant cultivars under stress. In fact, the results of these variables in salt-tolerant cultivars under salinity were similar to values under control conditions (Fig. 1B and C). This study found a clear positive effect on the water use efficiency (WUE) of salt-sensitive plants under stress (i.e., cultivar 1), while salt-tolerant cultivars retained similar values under stress and control conditions, indicating that were dealing better with the stress (Fig. 1D). This study also observed a negative effect of salt stress on photosynthesis (A) and on substomatic CO_2_ concentrations, although the intensity of changes was lower than for gs and E. Regarding cultivars, the differences among salt tolerant and salt sensitive cultivars were not significant for photosynthesis (Fig. 1E), but results showed higher substomatical CO_2_ concentrations for salt-tolerant cultivars, and again, similar to values obtained for non-stressed plants (Fig. 1F).

### Free amino acids

Once confirmed the tolerance and sensitivity of the selected cultivars at the physiological level, this study further investigated the level of essential metabolites. There is no description available in the literature regarding the behavior of the free amino acid pools under salt stress in *Brassica oleracea* var. *Italica*, so this study investigated the complete free amino acid profile in our plants under the studied conditions.

**Fig. 2:**
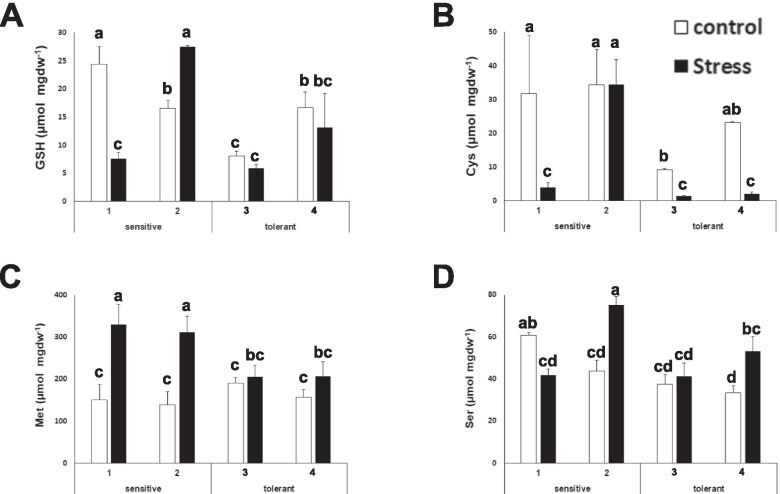
Glutathione, sulfur containing amino acids and serine determination. Glutathione (GSH) (**A**), cysteine (Cys) (**B**), methionine (Met) (**C**) and serine (Ser) (**D**) concentrations of salt-sensitive and salt-tolerant cultivars under control (white bars) and stress (black bars) conditions. Data with different letters differ significantly (*p* < 0.05), as determined by Duncan’s MRT test (*n* = 3). Scale bars are mean + SE

It has been shown that in response to abiotic stress the biosynthesis of sulfur containing amino acids may become limiting, specifically due to the requirement of cysteine for the biosynthesis of glutathione (GSH), which is required to cope with the oxidative stress induced by abiotic stress [31, 32]. GSH is also required for the biosynthesis of glucosinolates [33]. The total content of GSH and cysteine (Cys) was less in salt tolerant cultivars, under stress and control conditions (Fig. 2A and B). Methionine (Met) is a precursor of aliphatic glucosinolates [34]. The salt sensitive cultivars showed increased amounts of Met under salt stress, but the level was stable for the tolerant cultivars (Fig. 2C). This study did not find a distinctive pattern for serine (Ser) levels (Fig. 2D).

**Fig. 3:**
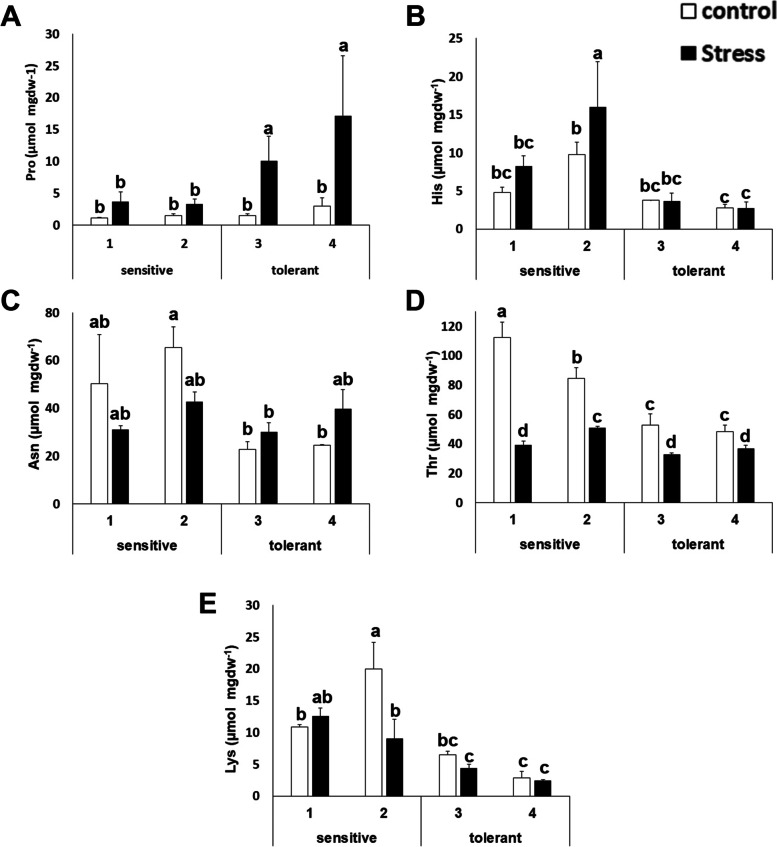
Amino acids with differential accumulation patterns between stress sensitive and stress tolerant cultivars. Proline (Pro) (**A**); histidine (His) (**B**); asparagine (Asn) (**C**); threonine (Thr) (D) and lysine (Lys) (**E**) concentrations of salt-sensitive and salt-tolerant cultivars under control (white bars) and stress (black bars) conditions. Data with different letters differ significantly (*p* < 0.05), as determined by Duncan’s MRT test (*n* = 3). Scale bars are mean + SE

Some amino acids can act as precursors for osmolytes or act as osmolytes themselves. Proline (Pro) is related to osmotic adjustment and has been described to accumulate in some *Brassicaceae* plants under drought stress [35]. This study also observed that proline accumulated upon salt stress. Interestingly, this accumulation was higher in salt tolerant cultivars (Figure 3A). On the other hand, histidine (His), asparagine (Asn), threonine (Thr) and lysine (Lys) levels in stressed and control conditions were lower for salt tolerant cultivars (Figure 3 B-E).

**Fig. 4:**
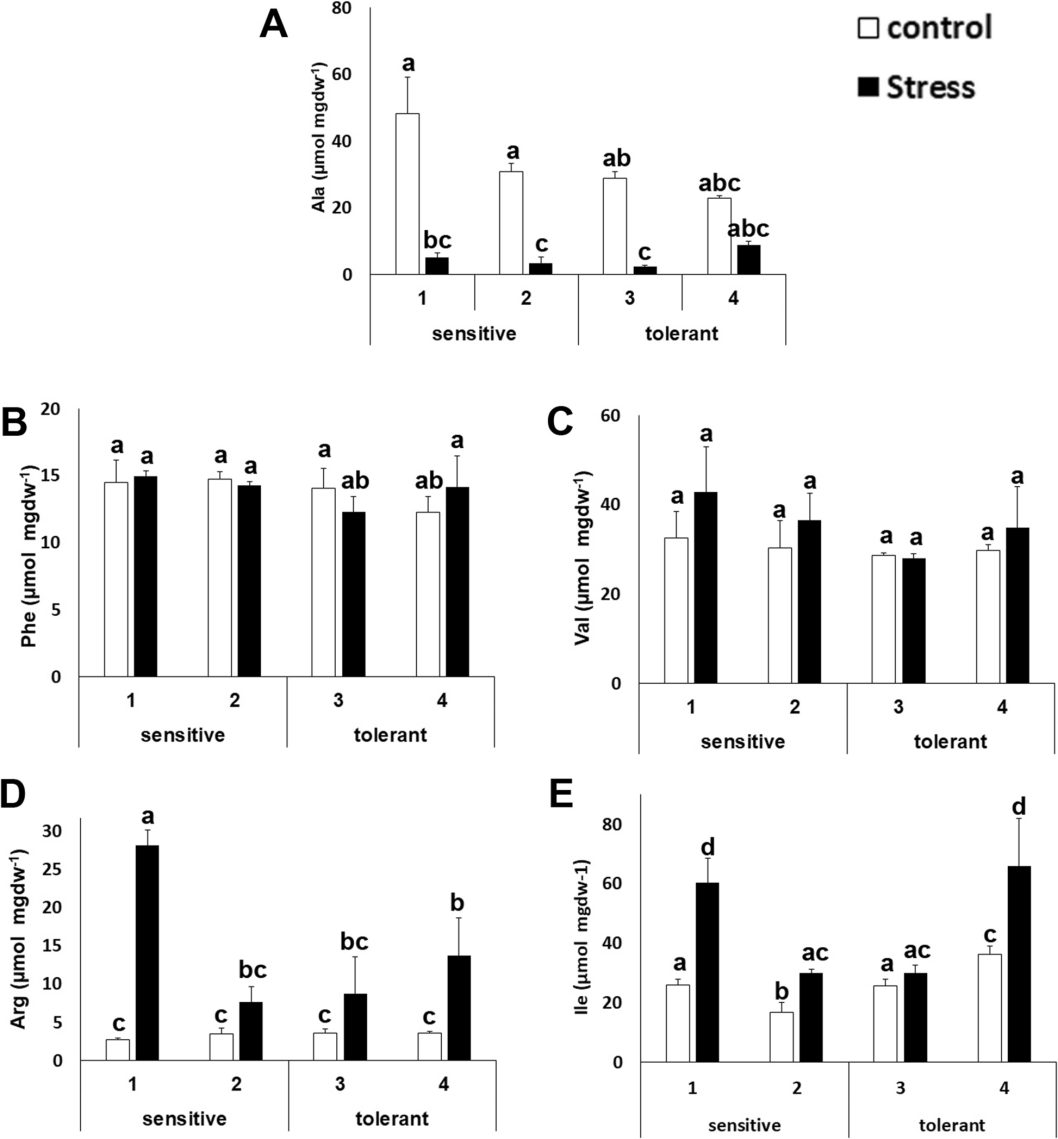
Amino acids with similar accumulation patterns between stress sensitive and stress tolerant cultivars. Alanine (Ala) (**A**); phenylalanine (Phe) (**B**); valine (Val) (**C**); arginine (Arg) (**D**) and isoleucine (Ile) (**E**) concentrations of salt-sensitive and salt-tolerant cultivars under control (white bars) and stress (black bars) conditions. Data with different letters differ significantly (*p* < 0.05), as determined by Duncan’s MRT test (*n* = 3). Scale bars are mean + SE

For other amino acids, this study did not observe differential patterns between salt-tolerant and salt-sensitive cultivars, but this study found a distinctive stress response. Alanine (Ala) decreased its concentration about 3- to 5-fold (Figure 4A). The phenylalanine (Phe) and valine (Val) concentrations stayed stable (Figure 4 B and C), while arginine (Arg) increased from 2- to 5-fold and isoleucine (Ile) increased between 50% to 3-fold (Figure 4 D and E).

### Hormone measurements

**Fig. 5:**
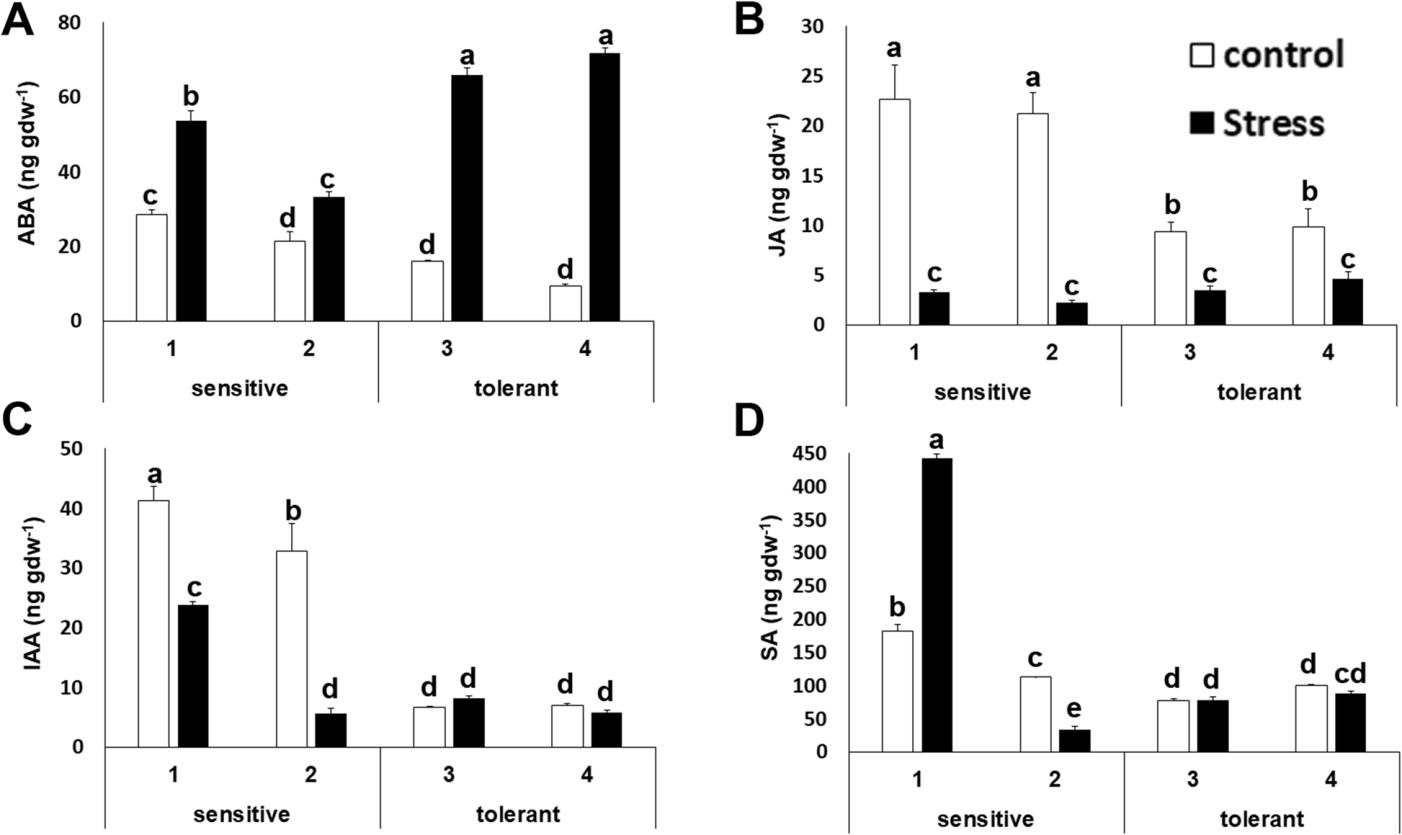
Hormone concentrations. Abscisic acid (ABA) (**A**); jasmonic acid (JA) (**B**); indoleacetic acid (IAA) (**C**) and salicylic acid (SA) (**D**) concentrations of salt-sensitive and salt-tolerant cultivars under control (white bars) and stress (black bars) conditions. Data with different letters differ significantly (*p* < 0.05), as determined by Duncan’s MRT test (*n* = 6). Scale bars are mean + SE

Plant hormones mediate salt stress responses and thus regulate plant growth adaptation [36]. This study investigated the differences in the hormone levels among cultivars under control and salt stress conditions. As expected, abscisic acid levels increased upon salt stress. Interestingly, salt tolerant cultivars showed decreased basal levels under control conditions, but exhibited a higher level upon salt stress, so the increase was 2- to 4-fold higher in salt tolerant cultivars (Figure 5A). Jasmonic acid levels decreased in all cases upon salt stress, but the levels were lower under basal conditions for salt tolerant cultivars and the decrease was about 3-fold less (Figure 5B). Indoleacetic and salicylic acid levels were also lower for salt tolerant cultivars (Figure 5 C and D).

### Sodium and potassium determination

**Fig. 6:**
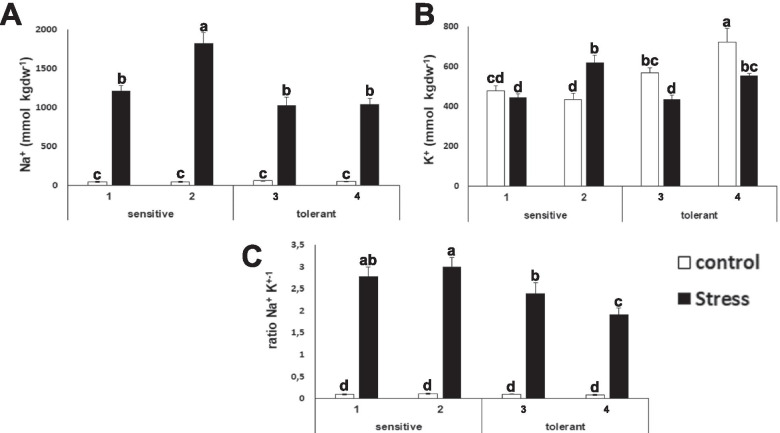
Ion content determination. Sodium content (Na^+^) (**A**), potassium content (K^+^) (**B**) and ratio Na^+^/K^+^ (**C**) concentrations of salt-sensitive and salt-tolerant cultivars under control (white bars) or stressed (black bars) treatments. Data with different letter differ significantly (*p*<0,05), as determined by Duncan’s MRT test (*n*=8). Scale bars are mean + SE

Sodium and potassium accumulation in leaves of control and stressed plants was determined. This study found that leaves from stress tolerant plants accumulate less sodium than leaves of sensitive plants (Figure 6A), and also less potassium (Figure 6B), but the Na^+^/K^+^ ratio is significantly lower for salt tolerant cultivars (Figure 6C), indicating that the loss of potassium is less than the uptake of sodium.

### Primary metabolite analysis

**Fig. 7:**
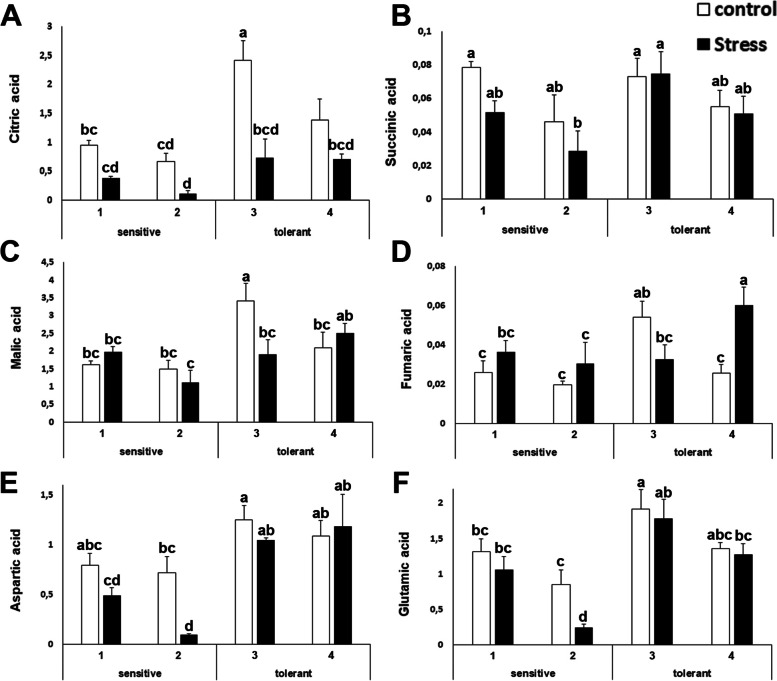
Concentration of primary metabolites related to the Krebs cycle. Citric acid (**A**); succinic acid (**B**); malic acid (**C**); fumaric acid (**D**); aspartic acid (**E**) and glutamic acid (**F**) concentrations of salt-sensitive and salt-tolerant cultivars under control (white bars) or stressed (black bars) treatments. The units are the area of the peak per mg of sample. Data with different letter differ significantly (*p*<0,05), as determined by Duncan’s MRT test (*n* = 4). Scale bars are mean + SE

This study found that intermediates of the Krebs cycle were present in higher amounts in salt tolerant plants under control or stressed conditions. This study could confirm this for citric, succinic, malic and fumaric acids (Figure 7A-D). This study also observed this pattern for aspartic and glutamic acid (Figure 7E-F). Both amino acids are the substrates of two anaplerotic reactions which feed the Krebs cycle, as aspartic acid is the precursor of oxalacetate and glutamate is both the product of the reaction of aspartic acid with α-ketoglutarate and the precursor of α-ketoglutarate.

**Fig. 8:**
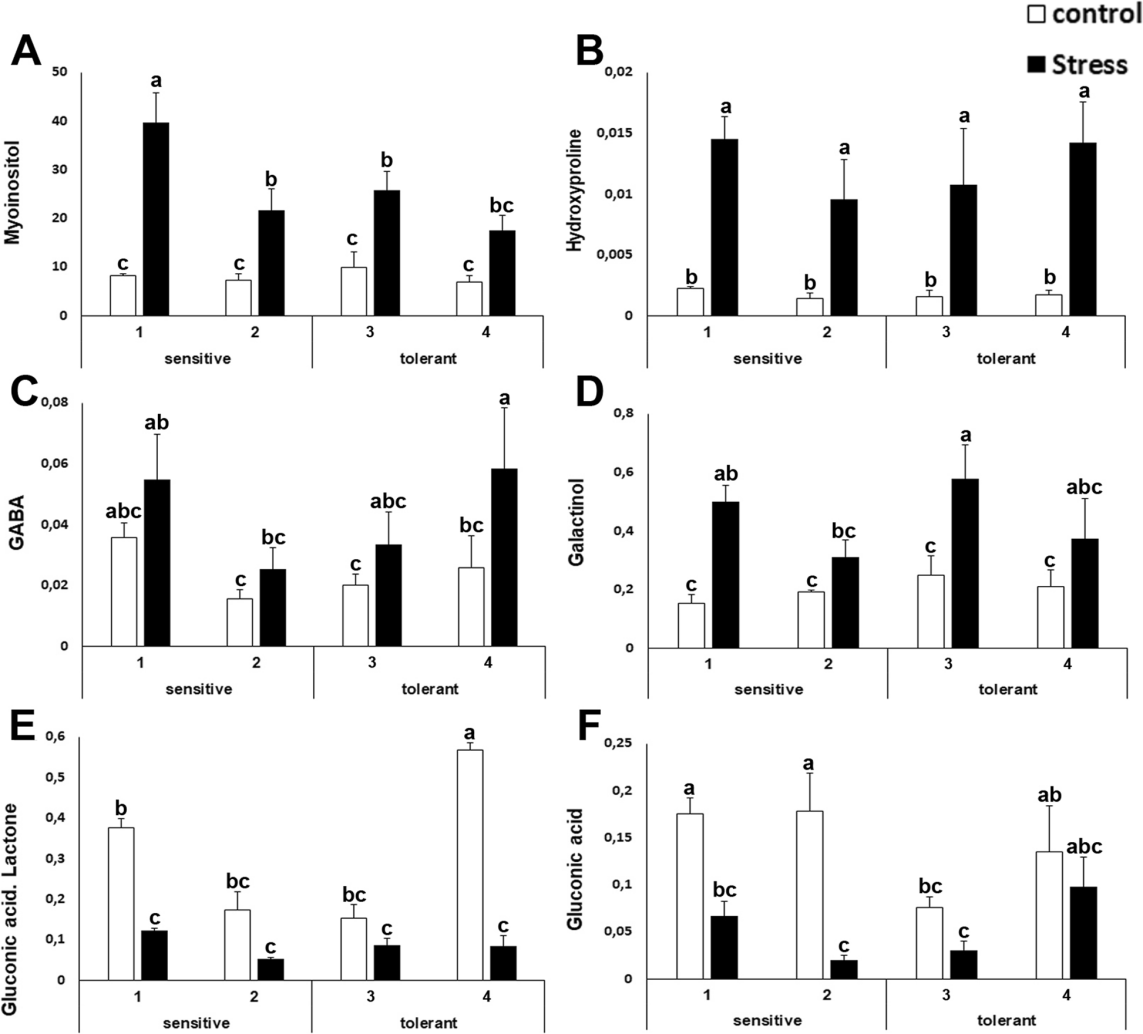
Concentration of primary metabolites altered by salt stress. Myoinositol (**A**); hydroxyproline (**B**); γ-aminobutyric acid (GABA) (**C**); galactinol (**D**); gluconic acid lactone (**E**) and gluconic acid (**F**) concentrations of salt-sensitive and salt-tolerant cultivars under control (white bars) or stressed (black bars) treatments. The units are the area of the peak per mg of sample. Data with different letter differ significantly (*p*<0,05), as determined by Duncan’s MRT test (*n*=4). Scale bars are mean + SE

This study also found that for some metabolites there was an effect due to salt stress, but no differential trend among sensitive and tolerant cultivars was observed. This study determined that upon salt stress the levels of myoinositol, hydroxyproline, γ-amino butyric acid (GABA) and galactinol increased 2- to 4-fold in leaves from salt stressed plants (Figure 8 A-D). On the other hand, gluconic acid and the lactone of gluconic acid decreased upon salt stress (Fig. 8E-F).

### Discussion

**Fig. 9:**
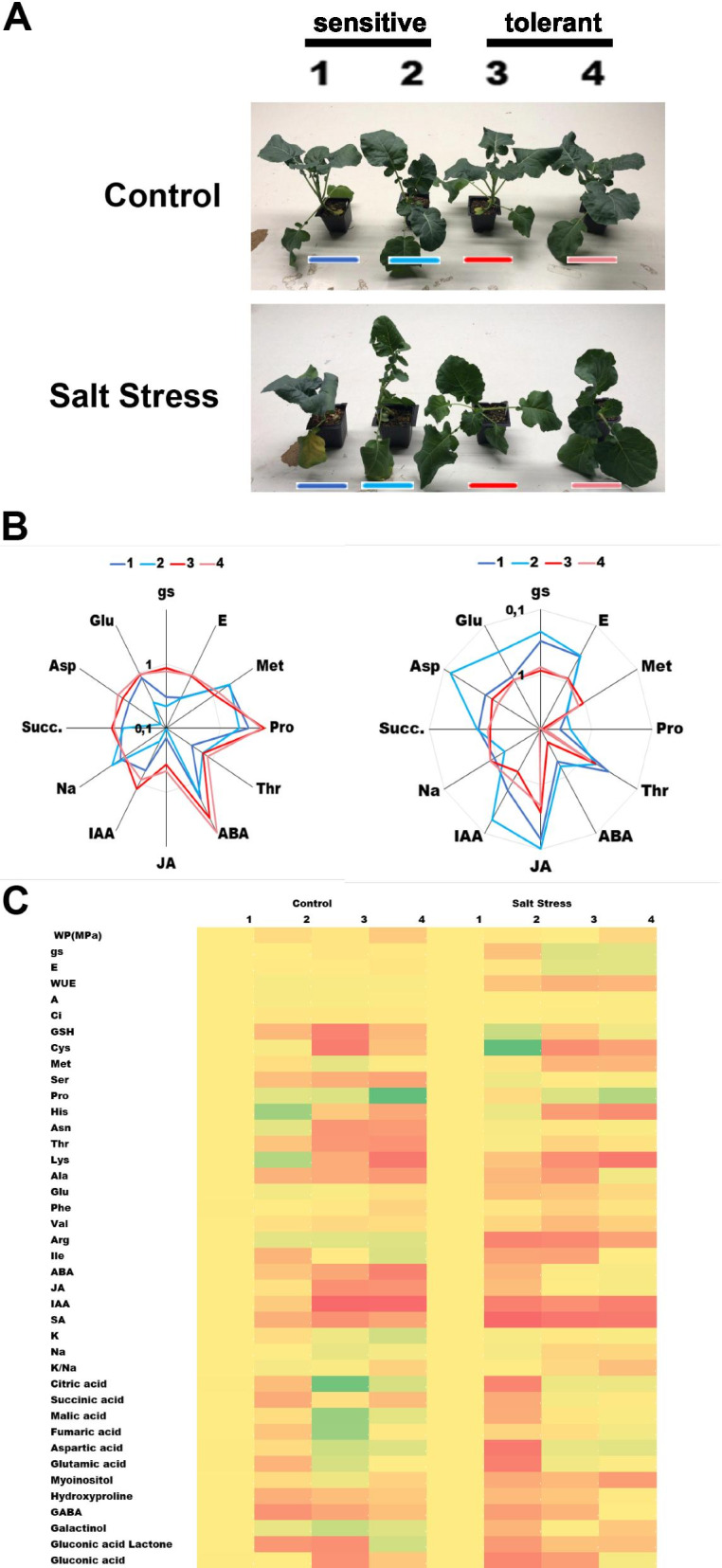
Summary of the main findings of this study. Representative plants of each cultivar under normal watering (upper panel) or after 6 days of salt stress (lower panel) (**A**). Radial diagrams of the ratio between stress/control concentration (left) or control/stress concentration (right). The tolerant lines are shown in shades of blue and the sensitive lines in shades of red. The values are represented in a decimal logarithmic scale (**B**). Heat map of all the results obtained in the present study. Green indicates higher normalized values, yellow average values while red indicates lower values (**C**)

This study has compared the effect of salt stress at the molecular and physiological level in two salt tolerant and two salt sensitive broccoli cultivars in order to identify distinctive traits among these cultivars that could predict the performance of uncharacterized cultivars under salt stress conditions and constitute limiting factors for salt stress tolerance. The main findings are summarized in Fig. 9.

A statistical analysis of all the physiological results indicated that the significant differences were linked to the stress sensitivity/tolerance of the cultivar, and not to individual cultivars (Table 1), thus validating our experimental design.

**Table 1:** Summary statistics of the general linear model and p-values (F) for the effect of treatments (control and salinity), degree of sensitivity to salinity (sensitive and tolerant), cultivars within sensitivities and the interaction between treatments and degree of sensitivity on broccoli plant performance. The numbers in bold indicate significant effects (*p*<0.05) and italics denote marginally significant effects (*p*<0.1)

Variable	Treatment (T) F(p)	Sensitivity (S) F(p)	Cultivar (V) F(p)	TxS F(p)
WP	**266.43 (<0.001)**	0.10 (0.751)	4.74 (0.358)	1.13 (0.358)
gs	**35.61 (<0.001)**	**9.45 (0.006)**	0.06 (0.945)	**4.51 (0.015)**
E	**42.08 (<0.001)**	**17.91 (0.001)**	0.04 (0.961)	**6.80 (0.003)**
WUE	**7.00 (0.015)**	*3.61 (0.070)*	0.61 (0.550)	*0.43 (0.092)*
A	**6.72 (0.017)**	2.66 (0.118)	0.88 (0.430)	1.07 (0.383)
Ci	**13.70 (0.001)**	*3.20 (0.086)*	0.64 (0.535)	**4.22 (0.016)**

As mentioned before all cultivars, both salt-sensitive and salt-tolerant were stressed by salinity, according to water potential measurements. However, salt tolerant cultivars were able to better withstand the stress, since values under stressed conditions were very similar to values obtained under control conditions for most of the parameters measured. In all samples tested, the water potential (Ψw) exhibited significant reductions in salt-stressed broccoli (Figure 1A). The validity of the experimental design was also confirmed by the increase in ABA (Figure 5A) and Pro (Figure 3A), which are standard indicators that the plant is responding to abiotic stress. The effect on stomatal conductance, transpiration and substomatal CO_2_ concentration was minor in the salt tolerant plants, probably reflecting that these cultivars were less affected by salt stress. Photosynthetic rates were less affected by salt stress; however, some tolerant cultivars had the same rates under control and salt stress conditions and no changes in transpiration were observed, in contrast to that observed in the salt sensitive plants (Figure 1).

Sulfur metabolism is pivotal in broccoli physiology. The synthesis of cysteine from serine and the subsequent biosynthesis of GSH is a key aspect of antioxidant defense. The serine acetyl transferase enzyme has been described as the main limiting factor for abiotic stress tolerance in several plants [31, 32]. Some reports confirm that GSH is the most important thiol involved in the prevention of oxidative damage in plants [37, 38]. In broccoli, salt stress induces an antioxidant response, which involves the enzymes involved in the regeneration of GSH [39]. In addition, sulfur and GSH are required for the biosynthesis of glucosinolates. This study observed that GSH, Cys and Met accumulation was lower in salt tolerant cultivars (Figure 2). It has been proposed that salt-tolerant species have higher glutathione content and higher redox states in comparison with salt-sensitive species [40–42]. Nevertheless, in the case of broccoli, it has been shown that glutathione increases after 24 hours of stress and then decreases, and that the main accumulation is observed in roots [43]. Therefore, the GSH and sulfur amino acid content in leaves may not be determinant for salt tolerance, and the observed low amounts may indicate that they are being used for the biosynthesis of other molecules (i.e glucosinolates) or are being accumulated in other tissues of the plant. Among others, Met is a substrate for the synthesis of various polyamines with important roles in stress tolerance [44, 45]. Therefore, it is likely that the lower level observed in salt tolerant plants indicates that Met is being recruited for the biosynthesis of molecules related to the stress response. Proline, in addition to its role as an amino acid, is an important osmolyte [46]. As expected, the proline content increased after salt stress [47]. In fact, broccoli is one of the vegetables capable of accumulating very high levels of proline [48]. This study found a distinctive pattern, as salt tolerant plants presented higher accumulation of proline, both under stress and control conditions, indicating that increased proline accumulation correlates with the salt tolerance of the plant (Figure 3A).

The hormone levels constitute a distinctive factor for salt tolerance or sensitivity in broccoli (Figure 5). Interestingly the ratio of the concentrations of ABA, JA and IAA from stress and control conditions were higher in salt tolerant cultivars, unveiling another distinctive trait among sensitive and tolerant cultivars (Figure 9 A and B). Salicylic acid levels were lower for tolerant plants. This result is surprising since in some crops it has been shown that external application of salicylic acid is able to alleviate the effects of salt stress [30].

Potassium is the major cation determining the intracellular ionic environment in plants [12, 49]. Our results indicate that salt tolerant cultivars accumulate less sodium in leaves, thus indicating that the role of the sodium extrusion systems is more limiting than sodium accumulation in the vacuole. There is another interesting outcome. The presence of salt in the soil has an osmotic effect, due to the ability of the ions to retain water. If tolerant cultivars have less sodium and less potassium, in principle, they must have less osmotic potential inside the cell. That means that tolerant cultivars should compensate this loss of ions with osmolytes. This study has found that tolerant cultivars accumulate more proline (Figure 3A). The current model for salt tolerance mechanism in brassica states that this family has a multiplicity of mechanisms, and among them, osmolyte accumulation, sodium extrusion and potassium retention [22]. Our experimental design further develops this model as it allows for the identification of the limiting factors for salt tolerance at least in early stages of development (5-6 weeks), as our experiments target to unveil the differences among sensitive and tolerant varieties. Taken together, the ability to extrude sodium, and to compensate for the loss of potassium with osmolytes (such as proline) may be a signature of tolerant cultivars, pointing to proline biosynthesis and sodium extrusion from roots or the cytosol (and not vacuolar accumulation) as the limiting factors for tolerance at the whole plant level, while the ability to accumulate potassium does not appear to be a distinctive factor. Therefore, this study has further defined the current model for salt stress tolerance in broccoli. In addition, this study has discovered that the energetic metabolism, at several steps, is also a distinctive trait. This is in agreement with the fact that the salt stress response is energetically costly, and thus explains why it is a self-destructive trait, gained often by selection, but frequently lost by reversal or extinction when the selection agent (salt stress) disappears [8]. Another valuable outcome of this study is that it has described that the levels of several amino acids and metabolites change upon stress, although their levels are not limiting for stress tolerance (Figure 4 and Figure 8). This contrasts to what is known for other crops like tomato, where the changes in the levels of amino acids are a distinctive trait for salt tolerance [50].

As mentioned before, from the bioenergetic point of view, the stress response is expensive. The biosynthesis of osmolytes and the maintenance of ion homeostasis requires large amounts of energy that must be diverted from other physiological processes, mostly related to plant development. This explains why under stress, plants slow or completely arrest their developmental program and yield decreases, which leads to important agricultural losses. Our metabolomic analysis pointed out that salt tolerant cultivars of broccoli have higher amounts of intermediates of the Krebs cycle, and of two substrates of anaplerotic reactions. The Krebs cycle is the main catabolic process for carbohydrates produced in the Calvin cycle. This study has shown before that physiological parameters related to gas exchange were also distinctive for salt tolerant plants and this holds true for broccoli as well. In addition, the anabolism and catabolism of carbohydrates, and thus, the ability to produce energy, is a main determinant for salt tolerance in broccoli, together with the increased osmolyte biosynthesis and sodium extrusion.

## Conclusion

This study has used a Greenhouse-based approach to determine differential traits at the physiological and molecular levels between salt-tolerant and salt-sensitive cultivars of broccoli at the initial stages of plant development (5-6 weeks). Our results indicate that the most distinctive trait for salt tolerance in broccoli is related to the ability to maintain photosynthesis and carbohydrate catabolism under salt stress, the levels of proline, the hormone concentration upon stress, and the ability to extrude sodium (Figure 9).

Taken together, this study proposes that the analysis of proline, hormone levels (i.e. ABA) or Krebs cycle intermediates (i.e. succinic acid) under salt stress in leaves of 5- to 6-week- old plants could be a fast and reliable method to screen for broccoli cultivars tolerant to salt stress. Our findings also may constitute the basis to develop biotechnological strategies, such as assisted or precision breeding techniques, to generate novel salt tolerant cultivars.

## Materials and methods

### Plant material and experimental conditions

Seeds of salt tolerant or salt sensitive cultivars were provided by ‘Sakata Seed Iberica’. The selection was based on field performance and confirmed by greenhouse tests under controlled salt stress. All are pre-commercial hybrid lines which are being developed by the company and are not available in the market yet.

The experimental design included two main factors: 1) stress level (control plants /salinity-stressed plants) and 2) cultivar (two previously characterized as salt sensitive and two as salt tolerant). For different experiments seeds were germinated in a Petri dish with filter paper. After five days, they were transferred to a substrate (50% kekkila peat, 25% perlite, 25% vermiculite) in TEKU cultivation trays, series PL 2838/24 with wells of 5.5 x 6 cm and volume of 149 mL. Greenhouse conditions were as follows: 16 h light/8 h dark (200 μmol m^−2^ s^−1^ of light intensity), at 24 ± 2°C and 70 ± 5% relative humidity. The experimental design consisted of an aleatory placement where each block was composed by 4 pots per tray and one plant per pot. Each experiment consisted in 3 individuals × 4 cultivars x 2 treatments (24 total plants) and was replicated 3 to 5 times. Plants were watered with Hoagland solution. After 5 weeks irrigation was kept (control plants) or salinity stress conditions were applied by watering with Hoagland solution plus 220 mM NaCl. Samples were taken or measures were performed after six days of stress treatment [51]. The same experimental design was used for determining the amino acid, hormone, ion and metabolite content. Samples were taken from the third youngest leave. In all cases, the number of samples per experiment (n) refers to biological replicates from different plants.

### Physiological measurements

Plants were grown under greenhouse conditions and stress was applied as described above. Physiological measurements were performed as described in Taibi et al., (2017) in the same leaves that were used for the rest of the determinations (i.e. third youngest leaf). The water potential (Ψw, -MPa) was measured with a Schölander pressure pump (model PMS-1000, PMS Instruments, Corvallis, OR, USA). For gas exchange measurements this study used a CIRAS-3 portable photosynthesis system (PP Systems, Amesbury MA, USA) under the following conditions: saturating light (1500 μmol of photons m^-2^ s^-1^), temperature of 25°C, ambient CO_2_ 390 μmol mol^-1^ CO_2_ and relative humidity of approximately 55%. The instantaneous determination of net CO_2_ assimilation -photosynthesis- (A, μmol CO_2_ m^-2^s^-1^), transpiration (E, mmol H_2_O^-2^s^-1^), stomatal conductance (gs, mol m^-2^s^-1^) and instantaneous water use efficiency (WUE, μmol CO_2_ mmol^-1^H_2_O) were determined in the same leaves in four replicates for each cultivar and condition.

### Amino acid analysis

One hundred mg of lyophilized leaf was grounded with a mortar and pestle in the presence of liquid nitrogen. The resulting powder was homogenized for 30 seconds with 2 mL of 2% citrate buffer pH 2 [31], boiled at 95^o^C for 12 minutes and centrifuged for five minutes at 13000 *g*. The supernatant was filtered through a 0,22-micrometer pore-size non-sterile filter. 1/10 dilutions of these extracts were injected into an automatic Beckman Gold amino acid analyzer. The analysis was carried out according to the protocol supplied by the manufacturer, using a system of ninhydrin and sodium citrate for detection.

### Hormone quantification

Plant hormones were quantified according to the method described in [52]. In brief, lyophilized tissue from the third youngest leaf (50 mg) was extracted in 2 mL of water after spiking with [^2^H_6_]-ABA, [^2^H_3_]-PA, dehydrojasmonic acid (DHJA), and [^13^C]-SA using a ball mill (MillMix20, Domel, Zelezniki, Slovenija). After the mechanical treatment, samples were centrifuged (4000 *g*, 10 min, 4°C) and the supernatants were collected. The pH was adjusted to 3 using acetic acid. Then extract was extracted using diethyl ether. This process was repeated twice. The upper layer was recovered and evaporated (Speed Vac, Jouan, Saint HerblainCedex, France). To resuspend the dry residue, we used 10% MeOH aided by gentle sonication. Then we passed filtered the solution (0,22μM Albet S.A., Barcelona, Spain), and injected it into a UPLC system (Acquity SDS, Waters Corp., Milford, MA) and analyzed as describe d[52]. Three biological replicates per cultivar and treatment were analyzed for each sampling time.

### Ion content determination

Ions were determined as described previously [53]. Briefly, samples of the third youngest leaf from 1-month-old plants (about 1 g) were dried at 70°C for 4 days. Dry weight was determined, and ions were extracted by a 30 min incubation in 1 mL of 0.1M HNO_3_ at room temperature. Then samples were centrifuged, and supernatant was diluted with 4 mL of milliQ water and filtered (0.22 μM pore-size). Sodium and potassium were measured in a plasma emission spectrophotometer (Shimadzu), as described [54].

### Primary metabolite analysis

Primary metabolite analysis was performed at the Instituto de Biología Molecular y Celular de Plantas (UPV-CSIC, Valencia, Spain) Metabolomics Platform by a method modified from that described by Roessner et al. 2000 [55]. 10 mg of leaves per sample were homogenized with liquid nitrogen and extracted in 1400 μL 100% methanol and 60 μL internal standard (0.2 mg ribitol in 1 mL of water). The mixture was extracted for 15 min at 70°C, then the extract was centrifuged for 10 minutes at 20,000 g. Supernatant was transferred to a glass vial and we added 750 μl of chloroform and 1,5 mL of milliQ water. After mixing and centrifuging at 20,000 g aliquots (0.15 mL) were taken and dried. Samples were analyzed as described in [55].

### Statistical design and analysis

The main treatment effects were analyzed by using the general linear model (ANOVA) considering three fixed factors: treatment (control and salinity), sensitivity degree to salinity (sensitive and tolerant) and cultivar (with four levels) as a factor nested within sensitivity degree. These analyses were performed with the SPSS v.25.0 statistical package (IBM SPSS Statistics for Windows, Armonk, NY, USA; IBM Corp.). The means were considered to be significantly different at *p* < 0.05 after Duncan's new multiple range test (MRT). In all cases, we used one factor analysis of Variance (ANOVA) and all groups were analyzed independently. For Figure 9B data in each line and treatment were normalized against the value of cultivar 1.

## Supplementary Information

**Additional file 1: Supplemental Figure 1.** The third leave of different plants (5 week old, 6 days of stress or control treatment) from salt-sensitive and salt-tolerant cultivars was cut and fresh weight and dry weight was determined under watered (white bars) and salt-stress (black bars) treatments (upper panel) and the ratio between stress and control conditions (lower panel). Data with different letters differ significantly (*p* < 0.05), as determined by Duncan’s MRT test (*n* = 4). Scale bars are mean + Statistical Error (SE).

## Data Availability

The datasets used and/pr analyzed during the current study are available from the corresponding author on reasonable request.
