# Supplementary figures and images for: Identification of distinctive physiological and molecular responses to salt stress among tolerant and sensitive cultivars of broccoli (Brassica oleracea var. Italica)
Source: BMC Plant Biol. 2021 Oct 25;21:488. doi: 10.1186/s12870-021-03263-4 (PMC8543863; doi:10.1186/s12870-021-03263-4)

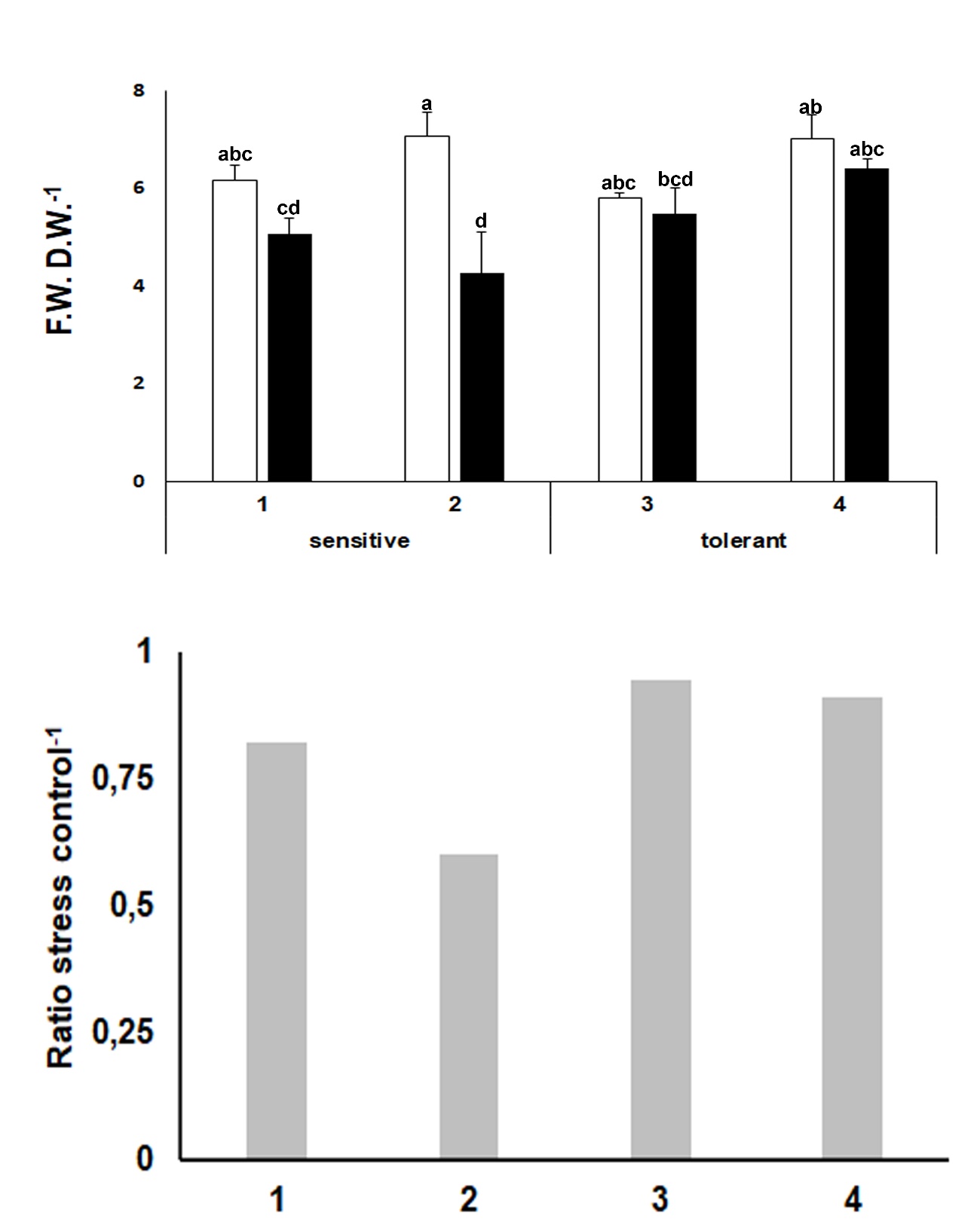

Supplement: Supplementary file 1 — Additional file 1: Supplemental Figure 1. The third leave of different plants (5 week old, 6 days of stress or control treatment) from salt-sensitive and salt-tolerant cultivars was cut and fresh weight and dry weight was determined under watered (white bars) and salt-stress (black bars) treatments (upper panel) and the ratio between stress and control conditions (lower panel). Data with different letters differ significantly (p < 0.05), as determined by Duncan’s MRT test (n = 4). Scale bars are mean + Statistical Error (SE). [file 12870_2021_3263_MOESM1_ESM.docx]
